# A case of isolated SMA dissection with failed initial conservative management

**DOI:** 10.1093/jscr/rjae789

**Published:** 2024-12-18

**Authors:** Salma Emara, Madison Kropp, Masoud Akbari, Kristin Cook

**Affiliations:** Department of Medicine, Rowan University School of Osteopathic Medicine, Stratford, NJ 08043, United States; Department of Surgery, Hackensack Meridian Palisades Medical Center 7600 River Road, North Bergen, NJ 07047, United States; Department of Surgery, Hackensack Meridian Palisades Medical Center 7600 River Road, North Bergen, NJ 07047, United States; Department of Surgery, Hackensack Meridian Palisades Medical Center 7600 River Road, North Bergen, NJ 07047, United States

**Keywords:** SMA dissection, spontaneous dissection, CT angiography, arteriography, endovascular repair

## Abstract

Isolated superior mesenteric dissection (ISMAD) is an uncommon condition, often diagnosed incidentally for presentations of acute abdominal pain. Early identification and treatment are crucial as complications such as bowel ischemia or vessel rupture can occur. There remain no established treatment guidelines, making surgical and endovascular indications controversial. A 60-year-old male presented with acute abdominal pain, and was diagnosed with ISMAD via computed tomography imaging. He was initially managed conservatively which progressed to worsening abdominal pain, hypertensive crisis, and hemoperitoneum on follow-up computed tomography angiography (CTA). A mesenteric angiogram revealed a pseudoaneurysm in the superior mesenteric artery (SMA) which was subsequently treated with coil embolization. The absence of long-term evidence on relapse rates questions the overall effectiveness of nonoperative therapy. Further research is needed to establish clear guidelines and to determine whether early intervention might be more advantageous in managing complications and preventing recurrence.

## Introduction

Isolated superior mesenteric artery dissection (ISMAD) is a rare but emerging vascular pathology, predominantly secondary to the widespread application of computed tomography (CT) imaging in the assessment of abdominal pain [1]. This condition usually occurs at 1.5–3 cm distal to the origin of the SMA, although it can also extend proximally, and typically presents with acute abdominal pain. Despite its increasing recognition, ISMAD remains a potentially fatal condition that lacks official evidence-based treatment guidelines.

The degree of vascular involvement and the patient’s general health can have a significant impact on the course of an SMA dissection, which can range from a self-limiting disease to a potentially fatal scenario. Generally, there are four possible outcomes from the dissection: (i) spontaneous resolution without long-term sequelae, (ii) spread to the entire vessel, (iii) formation of a dissecting aneurysm that joins the true lumen, or (iv) rupture of the vessel causing severe hemorrhage [2]. Diagnosis is usually confirmed by enhanced abdominal CT scans, which typically reveal a false lumen, an intramural hematoma, intimal flap, increased attenuation of fat surrounding the SMA, and/or mesenteric hematoma with hemorrhagic ascites [3]. While the exact pathogenesis of ISMAD remains unclear, potential causes include intimal or vasa vasorum tears leading to hemorrhage in the medial and adventitial layers of the vessel, with contributing factors such as hypertension, arteriosclerosis, and connective tissue disorders [2, 4].

Management varies depending on the patient’s hemodynamic stability and complications such as bowel ischemia or vessel rupture. Hemodynamically stable patients typically warrant conservative management [2]. Anticoagulation and/or antiplatelet therapy is typically used to prevent thrombus and embolus formation and to aid in recanalization of the true lumen; however, evidence supporting its effectiveness is largely observational [5]. Patients with signs of ischemia, progressive dissection, or aneurysmal dilation may require surgical or endovascular intervention, including thrombectomy, stent placement, or arterial bypass grafting [1, 6]. Understanding the natural history of the disease and indications for the three main treatment modalities are essential for optimizing patient outcomes.

## Case report

**Figure 1:**
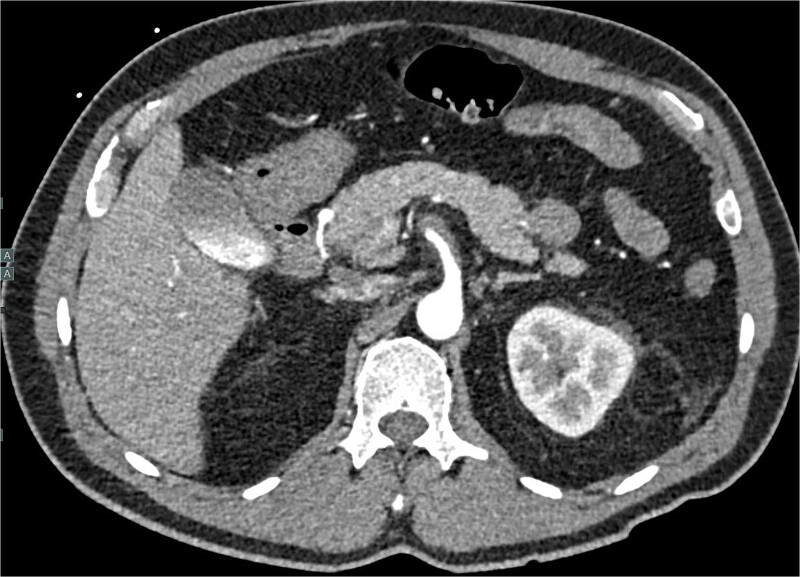
CTA abdomen and pelvis demonstrating 75% SMA stenosis secondary to SMA dissection with thrombosis of the false lumen.

A 60-year-old male with a past medical history of hypertension presented to our emergency department with 45 minutes of severe abdominal pain accompanied by nausea and vomiting. The patient’s presenting blood pressure was 192/130 mmHg with a heart rate of 77 bpm, and the remaining vitals were within normal limits. On physical examination, tenderness was noted in the epigastric region without peritoneal signs. A CT of the abdomen and pelvis revealed a partial thrombosis of the SMA, extending into some of the mesenteric branch arteries, with no evidence of bowel ischemia. The patient was started on a high-dose heparin drip. A same-day repeat CT angiogram further indicated 75% SMA stenosis secondary to SMA dissection with thrombosis of the false lumen (Fig. 1).

**Figure 2:**
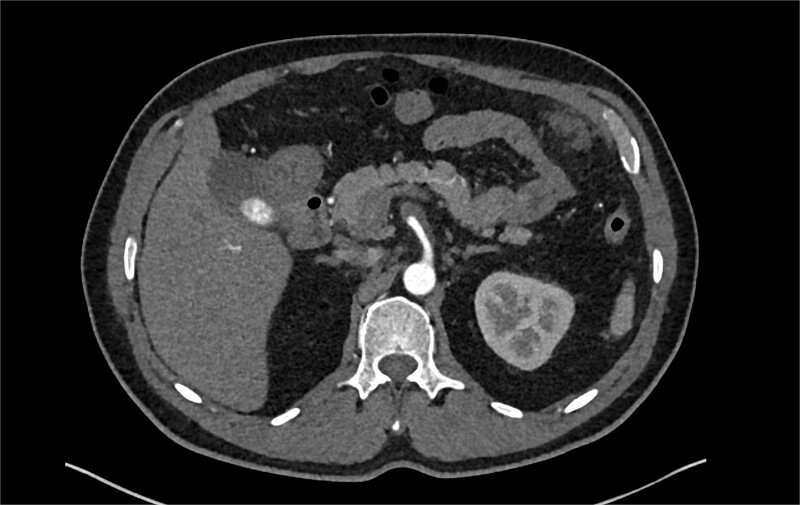
CTA demonstrating a more extensive SMA dissection extending into branch vessels. New hemoperitoneum most pronounced in the left abdomen with no active extravasation noted but probably arises from the left mesentery.

The patient was initially managed conservatively with blood pressure control and anticoagulation using Apixaban and aspirin. However, after one day of treatment, he experienced worsening abdominal pain and a hypertensive crisis with blood pressure reaching 228/113 mmHg. A follow-up CTA showed an extension of the SMA dissection extending into branch vessels, now associated with a small 1 cm aneurysm and hemoperitoneum (Fig. 2). Apixaban was stopped, and the patient was transitioned to a heparin drip, with an esmolol drip as needed for blood pressure control. As his symptoms improved, he was bridged back to apixaban and discharged on enoxaparin.

**Figure 3:**
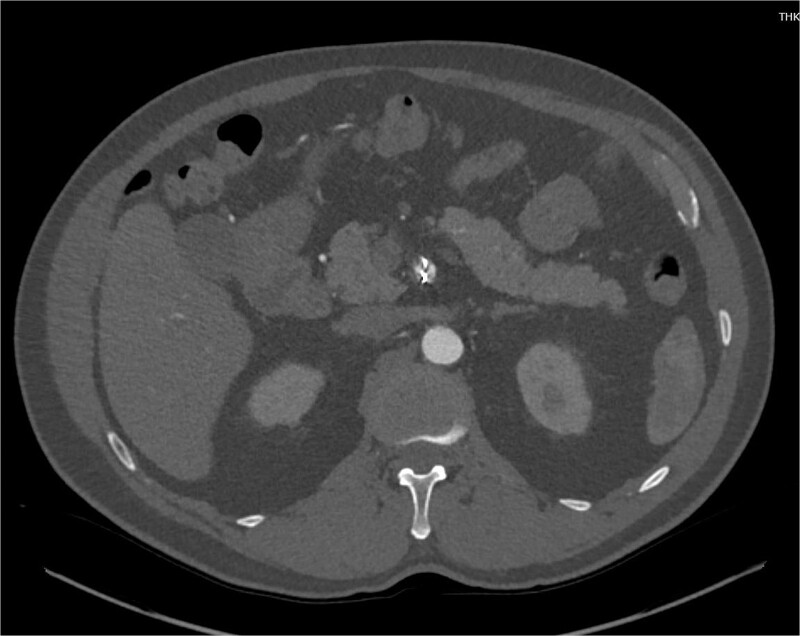
CTA demonstrating interval embolization of SMA at site of prior thrombus and dissection.

Despite medical management, the patient continued to have episodes of abdominal pain following discharge. One month after discharge, an outpatient mesenteric angiogram showed persistent SMA dissection and pseudoaneurysm. Coil embolization was performed to resolve the pseudoaneurysm. Following the procedure, the patient resumed anticoagulation therapy. The patient continued to follow outpatient studies with CTA indicating a normal SMA status post coil embolization of the pseudoaneurysm (Fig. 3).

## Discussion

This case of ISMAD emphasizes several key features. The patient initially presented with acute abdominal pain, a hallmark symptom of ISMAD, and was diagnosed via CT imaging. The dissection progressed despite initial conservative management, and worsening symptoms and imaging results led to a shift in therapeutic intervention. A meta-analysis of 727 patients with ISMAD found no benefit in treating symptomatic or asymptomatic individuals with medical therapy, while Peng *et al.* suggest initiating anticoagulation if there is a lack of clinical improvement [1, 2, 7–9]. There is no consensus on the timing for interventional therapy or surgery [8]. Park *et al.* and Zerbib *et al.* recommend intervention if abdominal pain persists for more than 7 days, while Jia et al recommend endovascular repair if symptoms continue for over 24 hours [10–12]. Endovascular stenting can be considered a primary treatment option for symptomatic patients with significant stenosis and who have not responded to conservative treatments [13]. In our patient, worsening symptoms prompted the use of coil embolization to address the pseudoaneurysm.

Morris *et al.* highlighted the value of CT imaging in the diagnosis, especially in detecting a false lumen. In our patient, his dissection extended into branch vessels and an associated hemoperitoneum, also consistent with previous reports that suggest conservative management may not be sufficient in cases involving complications such as thrombosis or aneurysmal dilation [2, 6].

Our patient also presented with a hypertensive crisis, which has been reported as a possible factor in the pathogenesis of ISMAD. Research completed by Bauersfeld (1947) and others however, has revealed that other factors, such as segmental arterial mediolysis or congenital connective tissue disorders may play a more primary role [14, 15]. Although the exact etiology remains unclear, the dissection in our patient’s case may have been influenced by his history of hypertension [2, 4].

Limitations to our study include a small sample size and insufficient long-term follow-up data. Clear guidelines for the management of ISMAD should be a main focus of future research, especially in situations where initial conservative management fails. In order to develop evidence-based guidelines that more reliably inform treatment decisions and to gain a deeper understanding of the natural history of ISMAD, there is a need for more extensive, long-term prospective trials.
